# Bofu-Tsu-Shosan, an Oriental Herbal Medicine, Exerts a Combinatorial Favorable Metabolic Modulation Including Antihypertensive Effect on a Mouse Model of Human Metabolic Disorders with Visceral Obesity

**DOI:** 10.1371/journal.pone.0075560

**Published:** 2013-10-09

**Authors:** Kengo Azushima, Kouichi Tamura, Hiromichi Wakui, Akinobu Maeda, Masato Ohsawa, Kazushi Uneda, Ryu Kobayashi, Tomohiko Kanaoka, Toru Dejima, Tetsuya Fujikawa, Akio Yamashita, Yoshiyuki Toya, Satoshi Umemura

**Affiliations:** 1 Department of Medical Science and Cardiorenal Medicine, Yokohama City University Graduate School of Medicine, Yokohama, Japan; 2 Department of Molecular Biology, Yokohama City University Graduate School of Medicine, Yokohama, Japan; Broad Institute of Harvard and MIT, United States of America

## Abstract

Accumulating evidence indicates that metabolic dysfunction with visceral obesity is a major medical problem associated with the development of hypertension, type 2 diabetes (T2DM) and dyslipidemia, and ultimately severe cardiovascular and renal disease. Therefore, an effective anti-obesity treatment with a concomitant improvement in metabolic profile is important for the treatment of metabolic dysfunction with visceral obesity. Bofu-tsu-shosan (BOF) is one of oriental herbal medicine and is clinically available to treat obesity in Japan. Although BOF is a candidate as a novel therapeutic strategy to improve metabolic dysfunction with obesity, the mechanism of its beneficial effect is not fully elucidated. Here, we investigated mechanism of therapeutic effects of BOF on KKAy mice, a model of human metabolic disorders with obesity. Chronic treatment of KKAy mice with BOF persistently decreased food intake, body weight gain, low-density lipoprotein cholesterol and systolic blood pressure. In addition, both tissue weight and cell size of white adipose tissue (WAT) were decreased, with concomitant increases in the expression of adiponectin and peroxisome proliferator-activated receptors genes in WAT as well as the circulating adiponectin level by BOF treatment. Furthermore, gene expression of uncoupling protein-1, a thermogenesis factor, in brown adipose tissue and rectal temperature were both elevated by BOF. Intriguingly, plasma acylated-ghrelin, an active form of orexigenic hormone, and short-term food intake were significantly decreased by single bolus administration of BOF. These results indicate that BOF exerts a combinatorial favorable metabolic modulation including antihypertensive effect, at least partially, via its beneficial effect on adipose tissue function and its appetite-inhibitory property through suppression on the ghrelin system.

## Introduction

Metabolic disorders with obesity have become a major medical problem associated with the development of hypertension, type 2 diabetes (T2DM) and dyslipidemia, and ultimately life-threatening cardiovascular and renal diseases. Since obesity with visceral fat is related to a variety of metabolic disorders and has a serious impact on the cost of health care, the treatment of obesity has become a critical issue. Hypertension is commonly present along with obesity, and it is often resistant to the typical antihypertensive treatment. Furthermore, hypertension with obesity is closely associated with the development of systemic organ damage and arteriosclerosis together with T2DM and dyslipidemia, and ultimately increases the morbidity of cardiovascular and renal diseases [1], [2], [3], [4]. Conversely, it has been reported that weight reduction contributes to a reduction in blood pressure in obese people [5], [6].

An effective anti-obesity treatment with a concomitant improvement in metabolic profile is essential in hypertension with visceral obesity. Although the standard treatment for obesity is a combination of diet and exercise therapy, it is often extremely difficult for obese people to reduce their body weight in this way compared with healthy people, in part because of their excessive appetite. Therefore, to prevent the development of obesity, various anti-obesity drugs and bariatric surgery have been developed as adjunct therapies of obesity in western medicine. However, anti-obesity drugs and bariatric surgery have only been able to help a limited number of severely obese people because of side effects and invasiveness of the procedure [7], [8].

Bofu-tsu-shosan (BOF) is one of oriental herbal medicine and is clinically available to treat obesity in Japan. BOF is composed of 18 crude herbal drugs (**Table 1**). In previous studies, BOF has been reported to exert its anti-obesity effect in obese patients as well as various obesity-model animals (i.e. MSG-obese mice, fructose-loaded rats, high fat-loaded mice and KKAy mice) [9], [10], [11], [12], [13], [14], [15]. However, the mechanism of its beneficial effects on visceral obesity with modulation of metabolic profile including blood pressure is not fully elucidated to date. In this study, to examine possible mechanisms involved in the beneficial effects of BOF on metabolic dysfunction with visceral obesity, we administered BOF to KKAy mice, which serve as a model of human metabolic disorders and spontaneously develop obesity with marked visceral fat, T2DM, dyslipidemia and hypertension under normal circumstances.

**Table 1:** Components of the BOF formula.

Crude drugs	Weight ratio (g)
Scutellariae radix	2.0
Glycyrrhizae radix	2.0
Platycodi radix	2.0
Gypsum fibrosum	2.0
Atractyloids rhizoma	2.0
Rhei rhizoma	1.5
Schizonepetae spica	1.2
Gardeniae fructus	1.2
Paeoniae radix	1.2
Cnidii rhizoma	1.2
Angelicae radix	1.2
Menthae herba	1.2
Ledebouriellae radix	1.2
Ephedrae herba	1.2
Forsythiae fructus	1.2
Zingiberis rhizoma	0.3
Kadinum	3.0
Natrium sulfuricum	0.7

BOF is composed of the 18 crude drugs. It has been shown that the main effective components of BOF were *shizonepetae spica, glycyrrhizae radix*, *forsythiae fructus* and *ephedra herba.*

## Materials and Methods

### Animals and Chronic BOF Treatment

This study was performed in accordance with the National Institutes of Health guidelines for the use of experimental animals. All of the animal studies were reviewed and approved by the animal studies committee of Yokohama City University.

Male KKAy mice (7 weeks old) were purchased from CLEA Japan (Tokyo, Japan). The animals were housed individually in an air-conditioned room (25°C) with a 12-hour light-dark cycle and were allowed free access to food and water. They were used in experiments after 2 weeks of acclimation. KKAy mice (9 weeks of age) were divided into two groups and fed a standard powdered diet (CE-2, the control group) and a powdered diet containing BOF (CE-2 containing 4.7% BOF, the BOF group) for 8 weeks. We determined the dosage of BOF and the period of BOF treatment by referring to the protocol of previous studies [9], [11], [12]. During the experiment, body weight and food intake were measured weekly and systolic blood pressure was measured by the tail-cuff method at the age of 9, 11, 13, 15 and 17 weeks. Mice were sacrificed in the fed state between 10∶00 and 14∶00 under anesthesia and the tissues were collected at the end of the experimental period (17 weeks of age).

BOF was used in the form of a powdered extract obtained by spray-drying the hot water extract of the mixture of the 18 herbal drugs as shown in Table 1. The study diet was prepared by mixing the powdered diet (CE-2; CLEA Japan) with BOF at a concentration of 4.7%. BOF and its ingredients were provided by Tsumura & Co. (Tokyo, Japan).

### Measurement of Blood Pressure

Systolic blood pressure and heart rate were measured by the tail-cuff method (BP- monitor MK-2000; Muromachi Kikai Co.) in a manner such that the blood pressure was measured without any preheating of the animals as described previously [16], [17], [18]. All measurements were performed once every two weeks between 10∶00–13∶00 during the experiment and at least eight values were taken for each measurement.

### Measurement of Rectal Temperature

Rectal temperature was measured using an electron thermistor equipped with rectal probe (BAT-12 Microprobe Thermometer, Physitemp Instruments Inc., New Jersey, USA). All experiments were performed between 9∶00–10∶00 when the mice were 17 weeks old.

### Blood Assays

Blood samples were obtained by cardiac puncture when the mice were sacrificed in the fed state. Enzymatic assay was used for the determination of plasma glucose, free fatty acids, total cholesterol, LDL cholesterol and HDL cholesterol (WAKO Pure Chemical, Osaka, Japan). Plasma adiponectin concentration was measured with a commercially available ELISA kit (Otsuka Pharmaceutical Co., Ltd, Tokyo, Japan).

### Histological Analysis

The epididymal white adipose tissues (WAT) were collected and fixed with 10% paraformaldehyde overnight and embedded in paraffin. Tissue sections were stained with hematoxylin and eosin for cell size determination. The adipocyte area was quantified using Image-Pro Plus software.

### Real-time Quantitative RT-PCR Analysis

Total RNA was extracted from epididymal WAT and interscapular brown adipose tissue (BAT) with ISOGEN (Nippon Gene), and the cDNA was synthesized using the Super Script III First-Strand System (Invitrogen). Real-Time Quantitative RT-PCR was performed with an ABI PRISM 7000 Sequence Detection System by incubating the reverse transcription product with the TaqMan Universal PCR Master Mix and designed TaqMan probe (Adiponectin: Mm_00456425_m1, PPAR-α: Mm_00440939_m1, PPAR-γ: Mm_01184322_m1, MCP-1: Mm_00441242_m1, TNF-α: 00443258_m1, IL-6: Mm_00446190_m1, UCP-1: Mm_01244861_m1, gp91*^phox^*: Mm_01287743_m1, p22*^phox^*: Mm_00514478_m1, p40*^phox^*: Mm_00476300_m1, p47*^phox^*: Mm_00447921_m1) (Applied Biosystems), essentially as described previously [19], [20]. The mRNA levels were expressed relative to those of the 18S rRNA control.

### Analysis of Acute Effect of BOF on Food Intake

To examine the acute effect of BOF on food intake, 13-week-old KKAy mice fed a standard diet were fasted for 24 hours and were then administered BOF (5000 mg/kg) dissolved in 1 mL of distilled water per 100 g of body weight via a stomach tube at 9∶00. This amount is equivalent to a one-day intake of BOF when the mice were allowed free access to a powdered diet containing 4.7% BOF. Measurement of the 24-hour food intake was started after the administration. As a control group, mice fasted for 24 hours were administered the same amount of distilled water in the BOF group and 24-hour food intake was measured similarly.

### Analysis of Acute Effect of BOF on Circulating Ghrelin Concentration

To examine the effect of BOF on plasma acylated-ghrelin level, 14-week-old KKAy mice a fed standard diet were fasted for 24 hours. In the BOF group, BOF (5000 mg/kg), dissolved in 1 mL of distilled water per 100 g of body weight, was administered to the mice via a stomach tube. In the control group, the same amount of distilled water was similarly administered to the mice. Two hours after the administration, the mice were sacrificed in the fasted state and blood samples were obtained. Blood samples were immediately centrifuged at 4°C, and the plasma was acidified with 1 mol/L HCL (1/10 of total volume). The plasma were stored at −80°C until use. The acylated-ghrelin level was determined using Active Ghrelin Enzyme-Linked Immunoassay Kit (Mitsubishi Kagaku Iatron, Inc., Tokyo, Japan).

### Statistical Analysis

All data are expressed as the means ± SEM. Differences between groups were analyzed by Student’s *t*-test or analysis of variance (2-way ANOVA) for multiple comparisons. Values of *P*<0.05 were considered statistically significant.

## Results

### Decrease in Food Intake and Body Weight in KKAy Mice Treated with BOF

**Figure 1:**
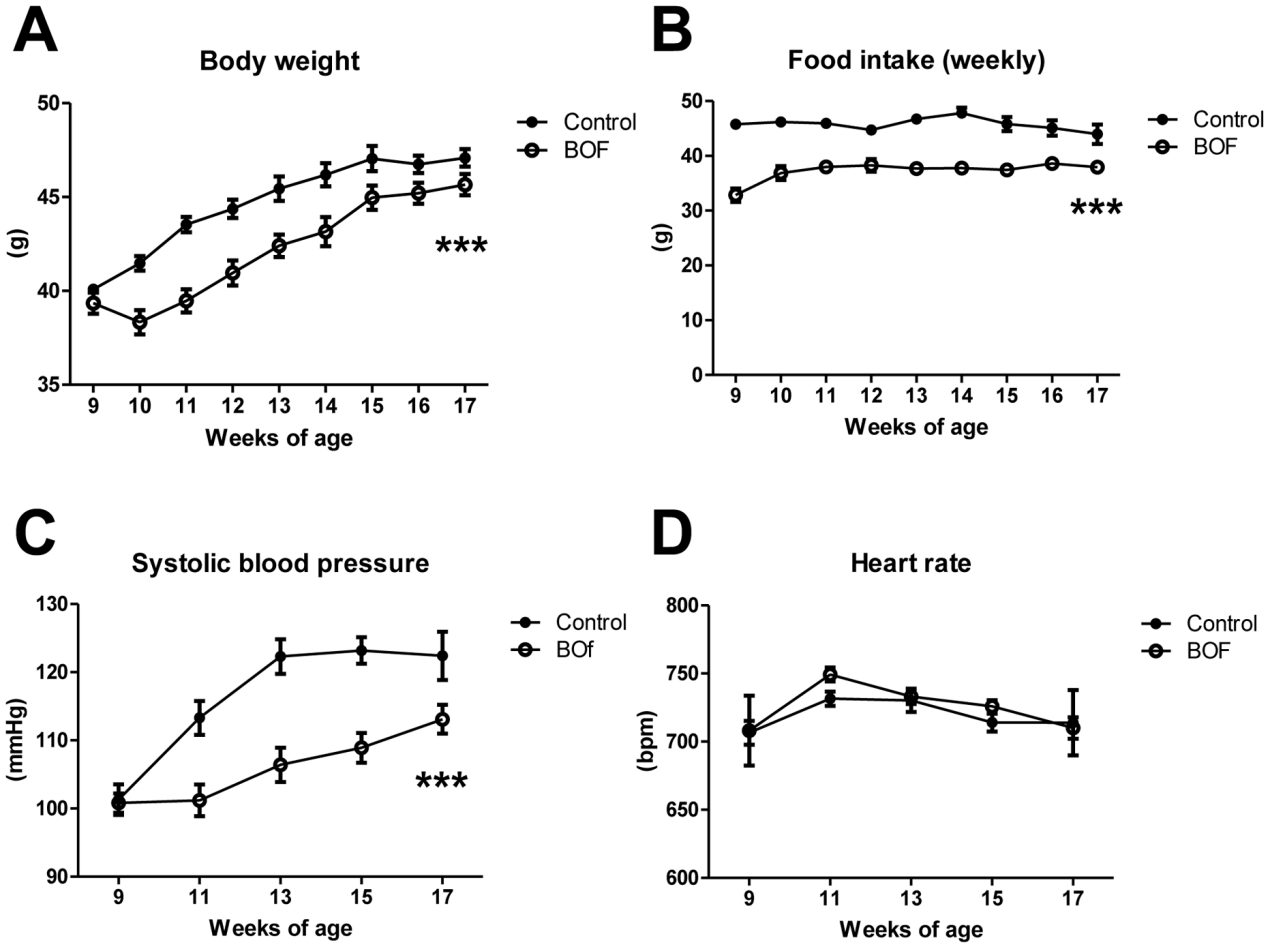
Chronic effects of BOF on body weight, food intake, systolic blood pressure and heart rate. KKAy mice (9 weeks old) were maintained on a standard powdered diet or a powdered diet containing 4.7% BOF for 8 weeks. (**A, B**) Body weight and food intake were measured weekly. (**C, D**) Systolic blood pressure and heart rate were measured once every two weeks by the tail-cuff method. Values are presented as the means ± SEM, ***P<0.001 by 2-way ANOVA, vs. control group (n = 6–9).

We firstly examined the effect of BOF on food intake and body weight of KKAy mice. The food intake in the BOF group was significantly decreased compared with the control group starting from the first week of the experiment and this effect was continued throughout the study period (2-way ANOVA *F* = 280.3, *P*<0.0001) (**Figure 1A**). The body weight change was significantly smaller in the BOF group than the control group during the entire experimental period (2-way ANOVA *F* = 81.04, *P*<0.0001) (**Figure 1B**).

### Decrease in Blood Pressure in KKAy Mice Treated with BOF

We next examined the effects of BOF on blood pressure and heart rate in KKAy mice. Systolic blood pressure and heart rate of KKAy mice were measured by the tail-cuff method at the age of 9, 11, 13, 15 and 17 weeks. Systolic blood pressure was significantly lower in the BOF group than the control group during the entire experimental period (2-way ANOVA *F* = 46.08, *P*<0.0001) (**Figure 1C**). On the other hand, there was no difference between the two groups in heart rate (2-way ANOVA *F* = 0.5660, *P* = 0.4642) (**Figure 1D**).

### Decrease in WAT Weight and Adipocyte Hypertrophy in KKAy Mice Treated with BOF

**Figure 2:**
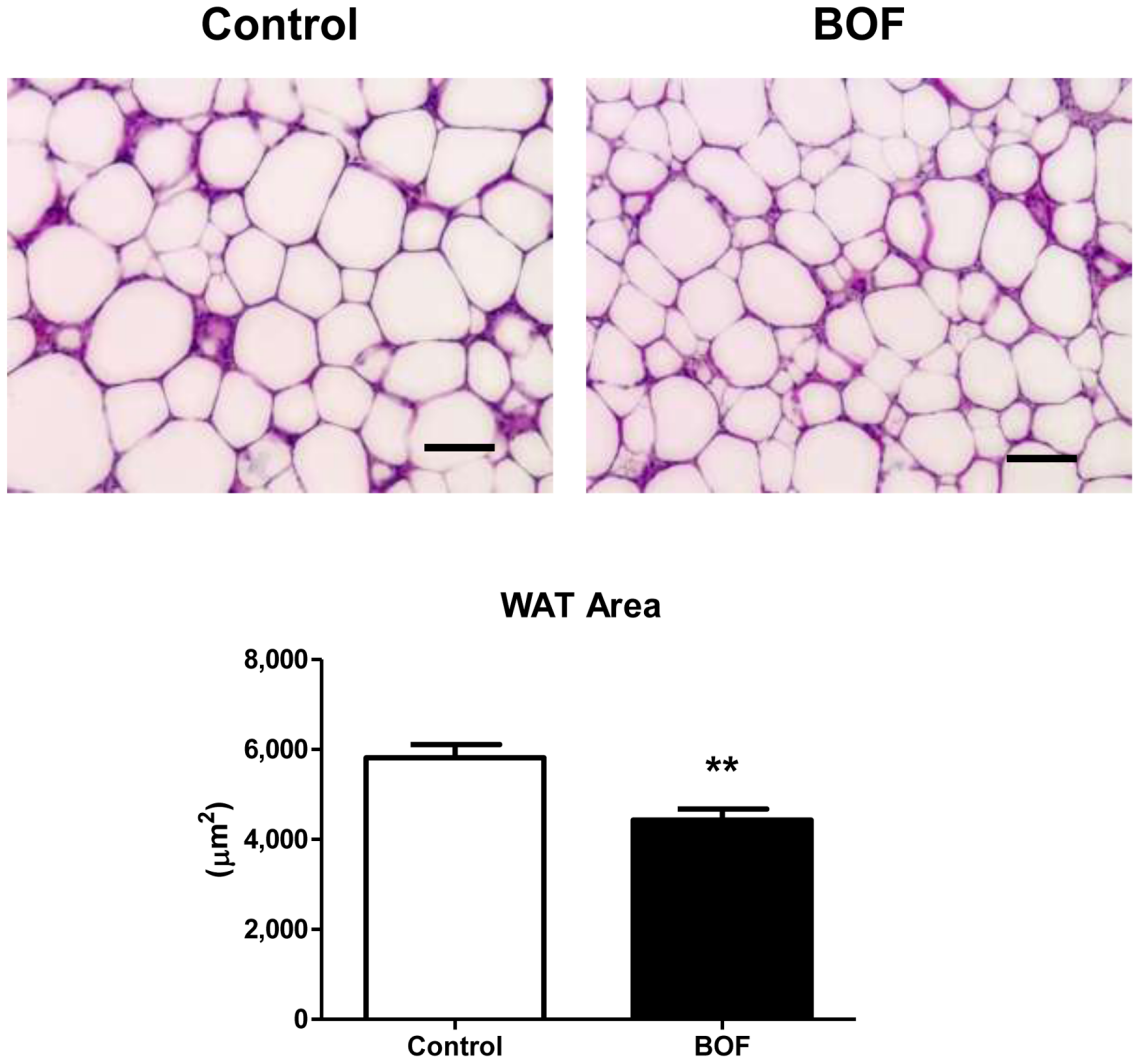
Chronic effects of BOF on adipocyte hypertrophy in epididymal WAT. The adipocyte size of WAT in each group was measured as described in ‘Materials and Methods’. Representative microscopic photos of WAT and the measurement of the WAT area are shown. Original magnification, ×200. Scale bar = 100 µm. Values are presented as the means ± SEM, **P<0.01 by Student’s *t*-test, vs. control group (n = 7–8).

Along with the decrease in body weight and blood pressure in the BOF group, the epididymal WAT weight was significantly decreased and the liver weight tended to be decreased compared with the control group (**Table 2**). Since the epididymal WAT weight was decreased in the BOF group, we examined whether the cell size of the epididymal WAT were different between the two groups. Indeed, the area size of epididymal WAT in the BOF group was significantly smaller than in the control group (area, 5816±293 vs 4433±241 µm^2^, *P* = 0.0034) (**Figure 2**). On the other hand, there was no significant difference in the mesenteric WAT weight or BAT weight between the two groups (**Table 2**).

**Table 2:** Effects of BOF on tissue weight and blood biochemical tests.

	Control	BOF	*P*-values
Epididymal adipose tissue (g)	1.248±0.049	1.081±0.046*	0.0285
Mesentery adipose tissue (g)	1.076±0.089	1.081±0.044	0.9644
Brown adipose tissue (g)	0.218±0.019	0.205±0.007	0.5133
Liver (g)	2.777±0.154	2.451±0.070	0.0900
Total-cholesterol (mg/dL)	118±8	119±3	0.9304
LDL-cholesterol (mg/dL)	8.9±0.5	4.3±0.3***	<0.0001
HDL-cholesterol (mg/dL)	56±5	65±3	0.1589
Free fatty acids (µEq/L)	196±31	251±39	0.3005
Glucose (mg/dL)	325±32	383±34	0.2467

Blood samples and tissues were corrected at the age of 17 weeks. Values are presented as the means ± SEM.

* *P*<0.05,

*** *P*<0.001 by Student’s *t*-test, vs control group (n = 7–8).

### Decrease in LDL (Low-density Lipoprotein) Cholesterol in KKAy Mice Treated with BOF

The smaller area of WAT in the BOF group prompted us to examine biochemical tests of glucose and lipid metabolism. As shown in **Table 2**, non-fasting blood glucose, free- fatty acids, total-cholesterol and HDL-cholesterol did not differ significantly between the two groups. However, LDL-cholesterol in the BOF group was significantly lower than in the control group (8.9±0.5 vs 4.3±0.3 mg/dL, *P*<0.0001) (**Table 2**).

### Amelioration of Adipocytokine Dysregulation in Epididymal WAT of KKAy Mice Treated with BOF

**Figure 3:**
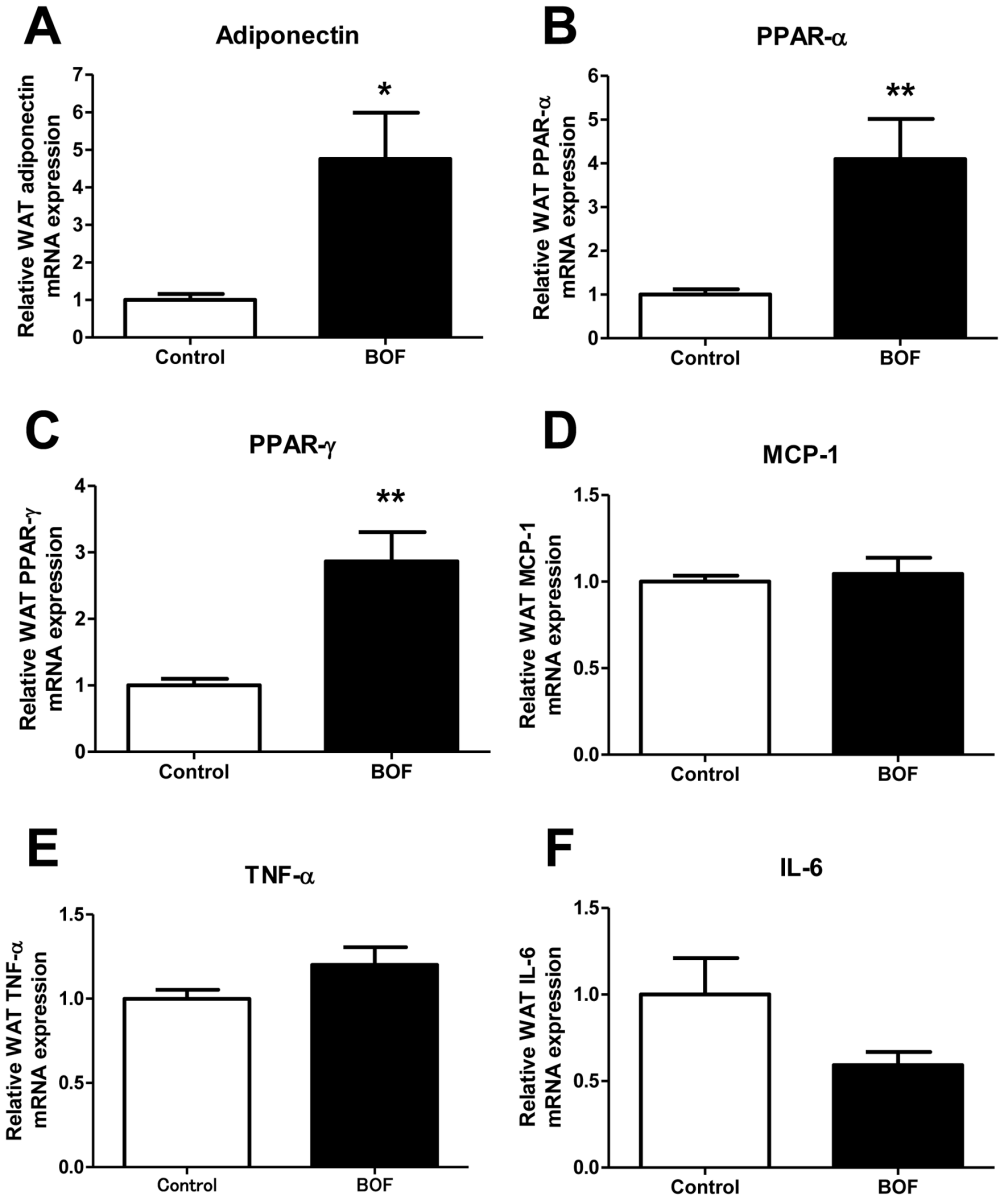
Chronic effects of BOF on adipocytokine dysregulation in epididymal WAT. (**A, B and C**) Expression of adiponectin and PPARs mRNA in WAT. (**D, E and F**) Expression of inflammatory cytokines mRNA in WAT. Values are presented as the means ± SEM, *P<0.05, **P<0.01 by Student’s *t*-test, vs. control group (n = 7–9).

To further investigate the effects of BOF on WAT, we examined adiponectin, peroxisome proliferator-activated receptors (PPARs) and inflammatory cytokine mRNA expression in epididymal WAT in KKAy mice. The result showed that the expression of adiponectin, PPAR-α and PPAR-γ mRNA was significantly elevated in WAT of the BOF group compared with the control group (**Figure 3A, 3B and 3C**). On the other hand, there were no apparent changes in the mRNA expression of NADPH oxidase (gp91*^phox^*, p22*^phox^*, p47*^phox^* and p40*^phox^*) in WAT of the BOF group (**Figure S1**). Furthermore, although no significant changes were observed in the expression of MCP-1, TNF-α mRNA (**Figure 3D, 3E**), the expression of IL-6 mRNA tended to be decreased in the BOF group (*P* = 0.0774) (**Figure 3F**).

### Increase in Circulating Adiponectin Level in KKAy Mice Treated with BOF

**Figure 4:**
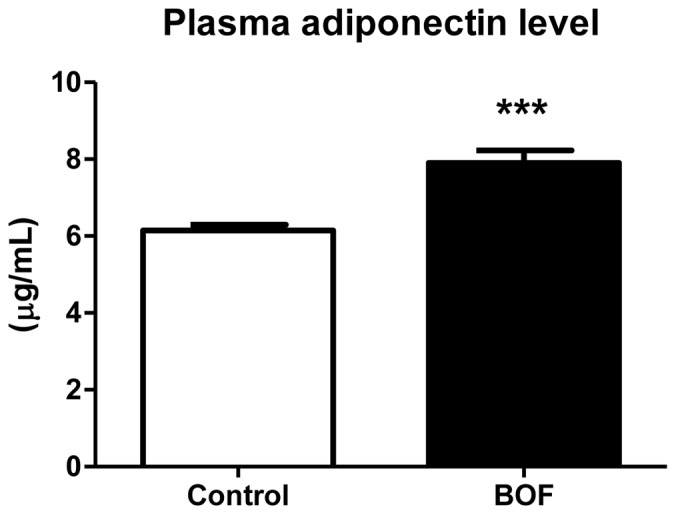
Chronic effects of BOF on circulating adiponectin level. Blood samples were corrected under the fed-state at the age of 17 weeks. Values are presented as the means ± SEM. ***P<0.001 by Student’s t-test, vs control group (n = 7–8).

The elevated adiponectin, PPAR-α and PPAR-γ mRNA levels in WAT of the BOF group prompted us to examine the effect of BOF on the circulating adiponectin level. As shown in **Figure 4**, plasma adiponectin concentration was significantly increased in the BOF group compared with the control group (6.1±0.4 vs 7.9±0.9 µg/mL, *P* = 0.0005).

On the other hand, a recent study revealed that hepatic up-regulation of PPAR-γ expression contributes to obesity-related hypertension via the sympathetic activation originating from the liver [21]. However, there was no significant change in hepatic PPAR-γ mRNA expression in the BOF group (**Figure S2**).

### Increase in Uncoupling Protein-1 (UCP-1) Gene Expression in BAT and Rectal Temperature in KKAy Mice Treated with BOF

**Figure 5:**
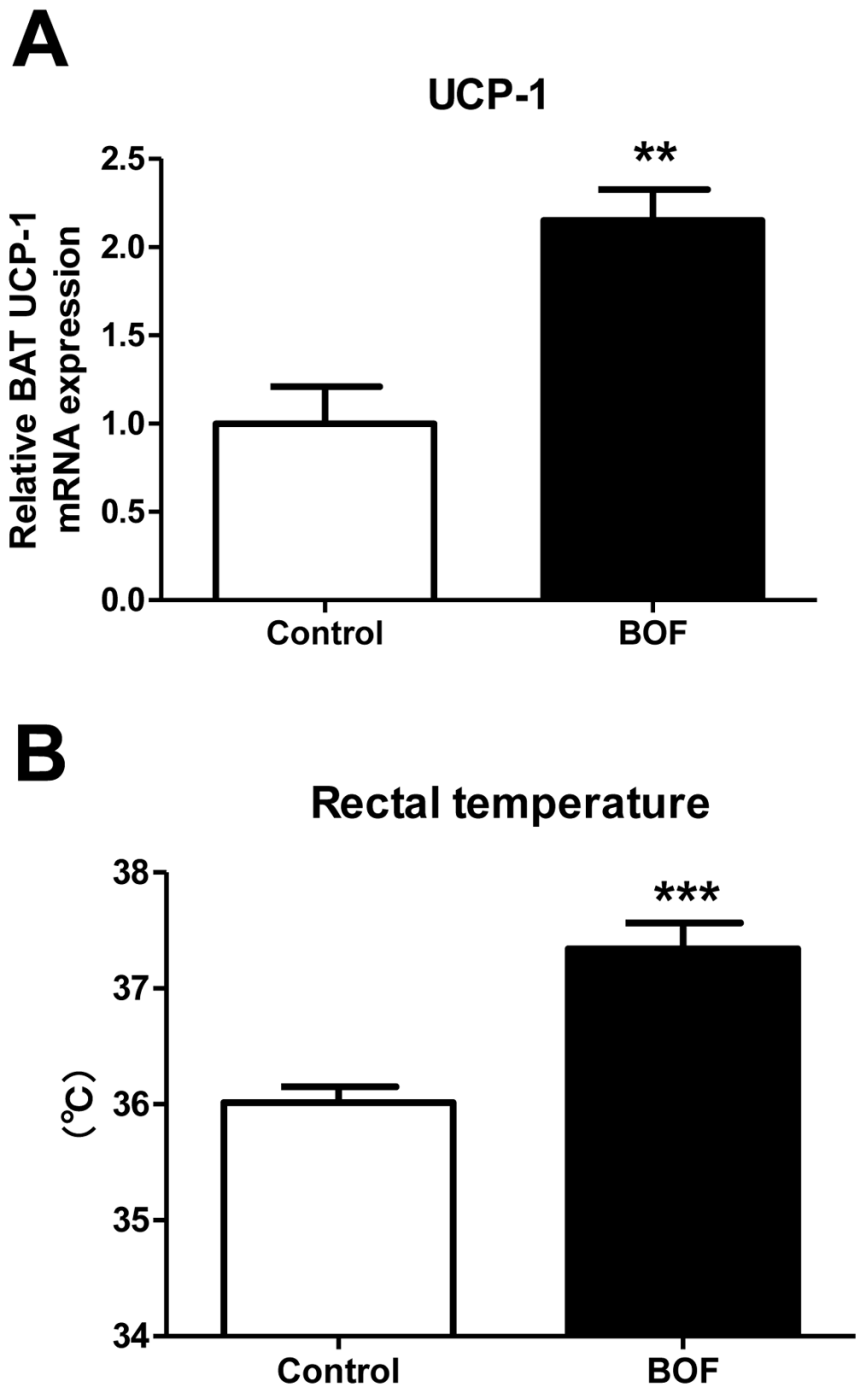
Chronic effects of BOF on BAT thermogenesis. (**A**) Expression of UCP-1 mRNA in BAT. (**B**) Rectal temperature was measured using an electron thermistor equipped with a rectal probe at the age of 17 weeks. Values are presented as the means ± SEM, **P<0.01, ***P<0.001 by Student’s *t*-test, vs. control group (n = 7–8).

To examine a role of activation of BAT thermogenesis in the anti-obesity effect of BOF, we examined the mRNA expression of the thermogenesis factor, UCP-1, in BAT and rectal temperatures in KKAy mice at the age of 17 weeks. Both the expression pattern of UCP-1 in BAT and the rectal temperature were significantly elevated in the BOF group compared with the control group (**Figure 5A and 5B**).

### Decrease in Food Intake and Circulating Ghrelin Concentration after Single Bolus Administration of BOF in KKAy Mice

**Figure 6:**
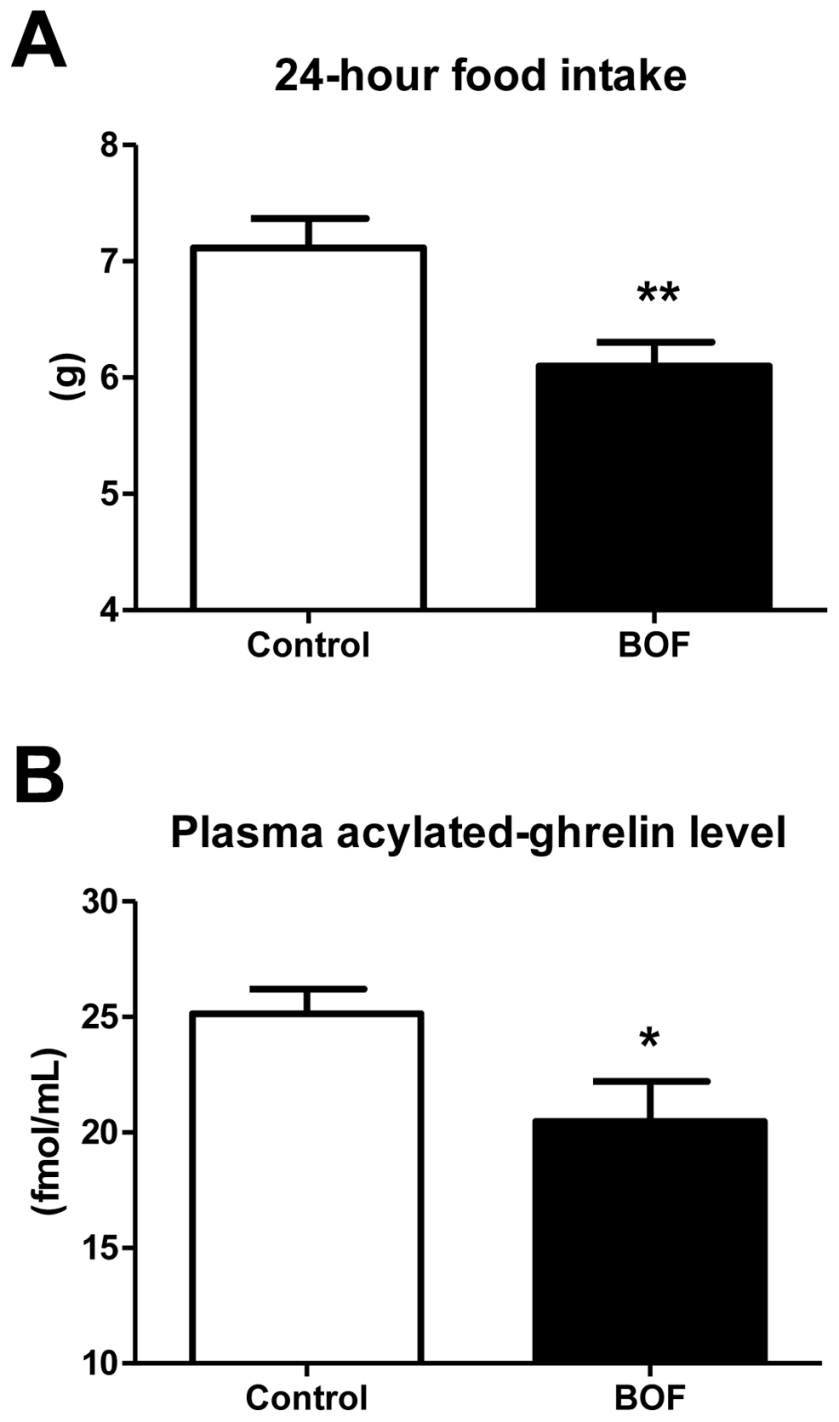
Acute effects of BOF on food intake and circulating active ghrelin concentration. (**A**) KKAy mice fed a standard diet were fasted for 24 hours and were administered BOF dissolved in distilled water via a stomach tube. After the single bolus BOF administration, 24-hour food intake was measured. (**B**) KKAy mice fed a standard diet were fasted 24 hours and were administered BOF dissolved in distilled water via a stomach tube. Two hours after the single bolus BOF administration, the mice were sacrificed in the fasted state, and blood samples were obtained. As a control group, we administered same amount of distilled water in the BOF group to mice under same conditions in each of the experiments. Values are presented as the means ± SEM, *P<0.05, **P<0.01 by Student’s *t*-test, vs. control group (n = 7–9).

We further investigated the acute effect of BOF on food intake in KKAy mice. We measured the 24-hour food intake after the single bolus administration of BOF. The 24-hour food intake in the BOF group was significantly decreased compared with the control group (7.1±0.3 vs 6.1±0.2 g, *P* = 0.0069) (**Figure 6A**). Based on this result, we hypothesized that BOF had an anorectic effect in the obese KKAy mice. Thus, we examined the acute effect of BOF on the circulating level of ghrelin, an orexigenic hormone. We ultimately found that the plasma level of acylated-ghrelin, the active form of ghrelin, was significantly decreased in the BOF group compared with the control group (25.1±1.1 vs 20.5±1.7 fmol/mL, *P* = 0.0355) (**Figure 6B**).

## Discussion

In the present study, we showed that 1) BOF persistently decreased food intake and body weight gain; 2) BOF consistently decreased blood pressure without affecting heart rate; 3) BOF decreased WAT weight and adipocyte hypertrophy, and ameliorated the adipocytokine dysregulation in WAT; 4) BOF increased UCP-1 mRNA expression in BAT and also the rectal temperature and 5) BOF decreased the short-term food intake and the plasma acylated-ghrelin level in KKAy mice, a model of human metabolic disorders with visceral obesity, T2DM, dyslipidemia and hypertension.

BOF treatment decreased adipocyte hypertrophy in WAT and elevated adiponectin, PPAR-α and PPAR-γ gene expression in WAT (**Figure 2** **,** **Figure 3**) as well as plasma adiponectin concentration (**Figure 4**). PPAR-γ is a critical regulator of adipocyte differentiation and is reported to activate adiponectin gene expression in WAT [22], [23], [24]. In addition, PPAR-α is suggested to directly enhance the adiponectin gene expression in WAT [25], [26]. Furthermore, adiponectin is reported to play a protective role against hypertension [27], [28], [29], and adiponectin replenishment is shown to suppress salt-induced hypertension in mice [30].

Importantly, chronic BOF treatment decreased not only body weight but also food intake in the KKAy mice (**Figure 1**). Several studies have shown that food restriction causes a reduction of visceral fat and elevated adiponectin and PPARs mRNA expression in WAT [26], [31], [32]. Caloric restriction in spontaneously hypertensive rats prevented hypertension through an increase in the circulating adiponectin level [33]. These results support that the activation of adiponectin gene expression in WAT and the elevated circulating adiponectin concentration, at least partially, play a role in the BOF-mediated blood pressure lowering via decrease in food intake in KKAy mice.

In addition, the chronic BOF treatment elevated UCP-1 mRNA expression in BAT and also the rectal temperature (**Figure 5**), thereby suggesting the activation of BAT thermogenesis by BOF. UCP-1 is specifically expressed in BAT and uncouples mitochondrial oxidative phosphorylation by bypassing the electrochemical gradient across the inner membrane from the F1-ATPase and thereby consumes energy as heat [9]. Thus, the elevated rectal temperature, induced by activating BAT thermogenesis may contribute to the efficient suppression of adipocyte hypertrophy in WAT.

Since BOF decreased food intake (**Figure 1** **,** **Figure 6**), one of anti-obesity properties of BOF is suggested to directly inhibit the activity for food intake. Ghrelin, an orexigenic hormone secreted mainly from the stomach, plays an important role in the regulation of food intake [34]. Ghrelin is reported to cause a significant increase in food intake by exerting a potent appetite-stimulating effect by altering orexigenic neuropeptides in the hypothalamus [35], [36], [37], [38], [39], [40]. Thus, we hypothesized that BOF exerts a potent appetite-inhibitory effect possibly via suppression of the ghrelin system. We demonstrated that the acute BOF administration significantly decreased the 24-hour food intake with a concomitant reduction of circulating concentration of activated ghrelin (**Figure 6**). This is the first report showing that BOF exerts an appetite-inhibitory effect via its suppression on the ghrelin system.

However, further investigation is necessary to determine the effective components of BOF and organs on which BOF acts. In addition, a limitation of the present study is that we have not examined a possible effect of BOF on markers of insulin resistance in KKAy mice. A previous study showed that the treatment with BOF increased plasma insulin levels in spite of suppression of body weight gain and decrease in visceral fat weight in KKAy mice, thereby suggesting an improvement in pancreatic insulin secretion by BOF [12]. Further studies such as glucose tolerance test and insulin tolerance test are needed to examine possible effects of BOF on pancreatic insulin secretion and insulin resistance in KKAy mice. Furthermore, other factors such as a diuretic effect and/or an inhibitory effect on sympathetic nerve activity, in addition to a decrease in food intake via appetite suppression, may be involved in the BOF-mediated blood pressure lowering, and these issues should be also addressed by further studies.

**Figure 7:**
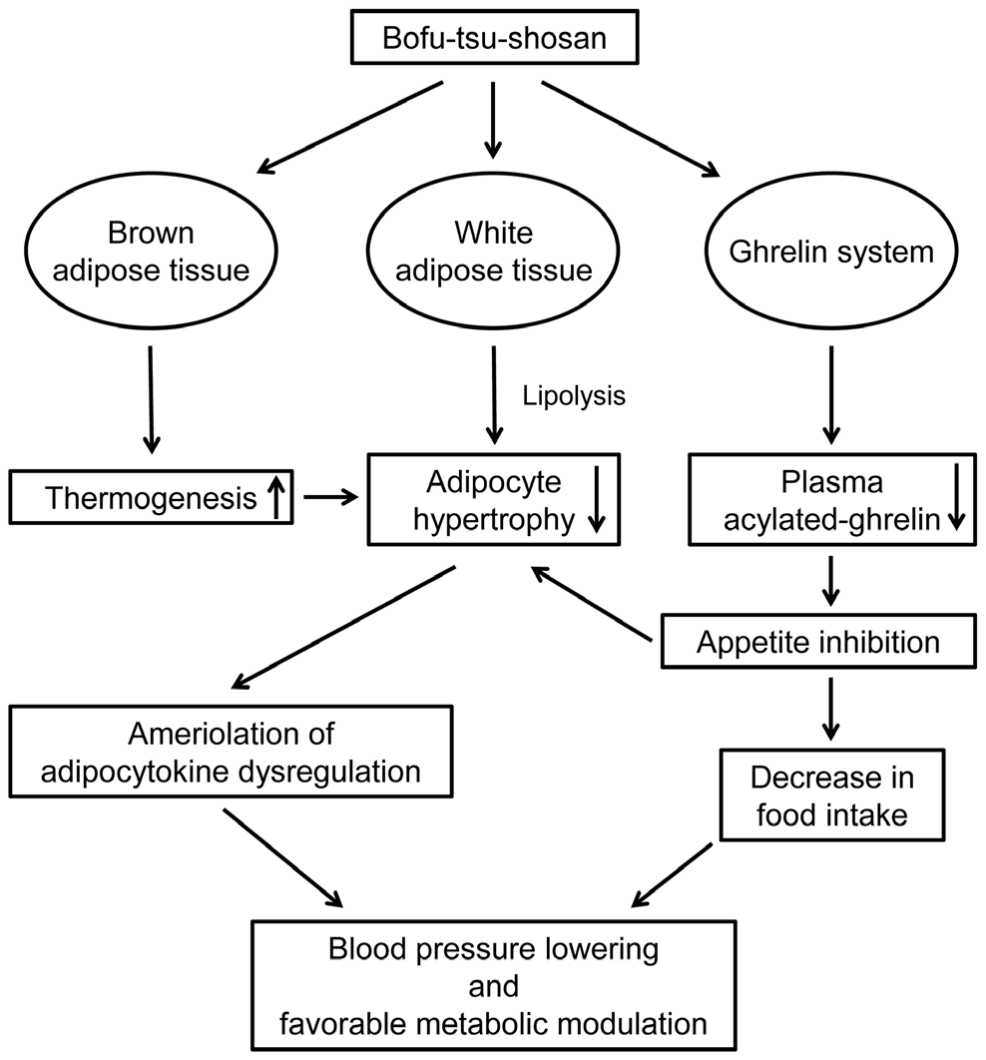
Graphical representation of the mechanisms of beneficial effects induced by BOF. BOF acts on adipose tissue to decreases adipocyte hypertrophy in WAT by activation of BAT thermogenesis and amelioration of adipocytokine dysregulation. BOF probably acts on the ghrelin system to exert an appetite inhibitory effect. The improvement in adipose tissue function by amelioration of adipocytokine dysregulation and the reduction of food intake by appetite inhibition contribute to the blood pressure lowering effect as well as favorable metabolic modulation by BOF in KKAy mice.

Collectively, from the results of present study, we present a schema that would explain the mechanisms of the combinatorial favorable metabolic modulation including antihypertensive effect mediated by BOF (**Figure 7**). BOF acts on adipose tissue to decrease adipocyte hypertrophy in WAT by activation of BAT thermogenesis and amelioration of adipocytokine dysregulation. BOF probably acts on the ghrelin system to exert an appetite inhibitory effect. The improvement in adipose tissue function by amelioration of adipocytokine dysregulation and the reduction of food intake by appetite inhibition may contribute to the blood pressure lowering effect as well as favorable metabolic modulation by BOF in KKAy mice. Although clinical trials in human are needed, it is possible that a combination therapy of BOF and western medicine, such as anti-obesity drugs, bariatric surgery and renin-angiotensin system inhibitors, may be able to treat pathological visceral obesity with metabolic disorders more effectively than at present.

## Supporting Information

Figure S1 Chronic effects of BOF on NADPH oxidase expression in epididymal WAT. Expression of NADPH oxidase subunits mRNA (**A**, gp91*^phox^*; **B**, p22*^phox^*; **C**, p47*^phox^* and **D**, p40*^phox^*) in WAT. Values are presented as the means ± SEM, *P<0.05 by Student’s *t*-test, vs. control group (n = 7–9).
(TIF)

Figure S2 Chronic effects of BOF on PPAR-γ expression in liver. Expression of PPAR-γ mRNA in liver. Values are presented as the means ± SEM, *P<0.05 by Student’s *t*-test, vs. control group (n = 8).
(TIF)
