# Supplementary figures and images for: Bofu-Tsu-Shosan, an Oriental Herbal Medicine, Exerts a Combinatorial Favorable Metabolic Modulation Including Antihypertensive Effect on a Mouse Model of Human Metabolic Disorders with Visceral Obesity
Source: PLoS One. 2013 Oct 9;8(10):e75560. doi: 10.1371/journal.pone.0075560 (PMC3794018; doi:10.1371/journal.pone.0075560)

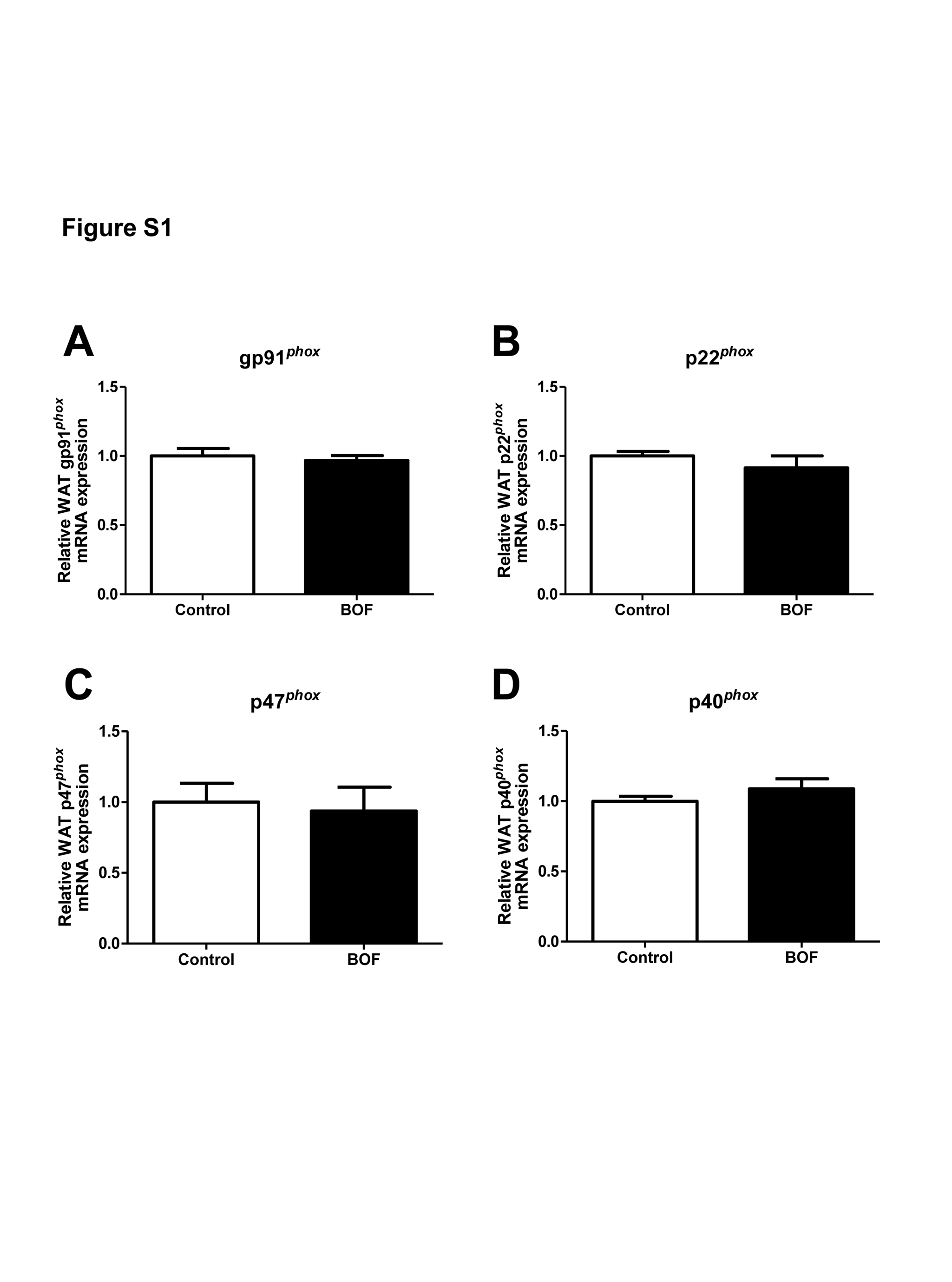

Supplement: Figure S1 — Chronic effects of BOF on NADPH oxidase expression in epididymal WAT. Expression of NADPH oxidase subunits mRNA (A, gp91phox; B, p22phox; C, p47phox and D, p40phox) in WAT. Values are presented as the means ± SEM, *P<0.05 by Student’s t-test, vs. control group (n = 7–9). (TIF) [file pone.0075560.s001.tif]

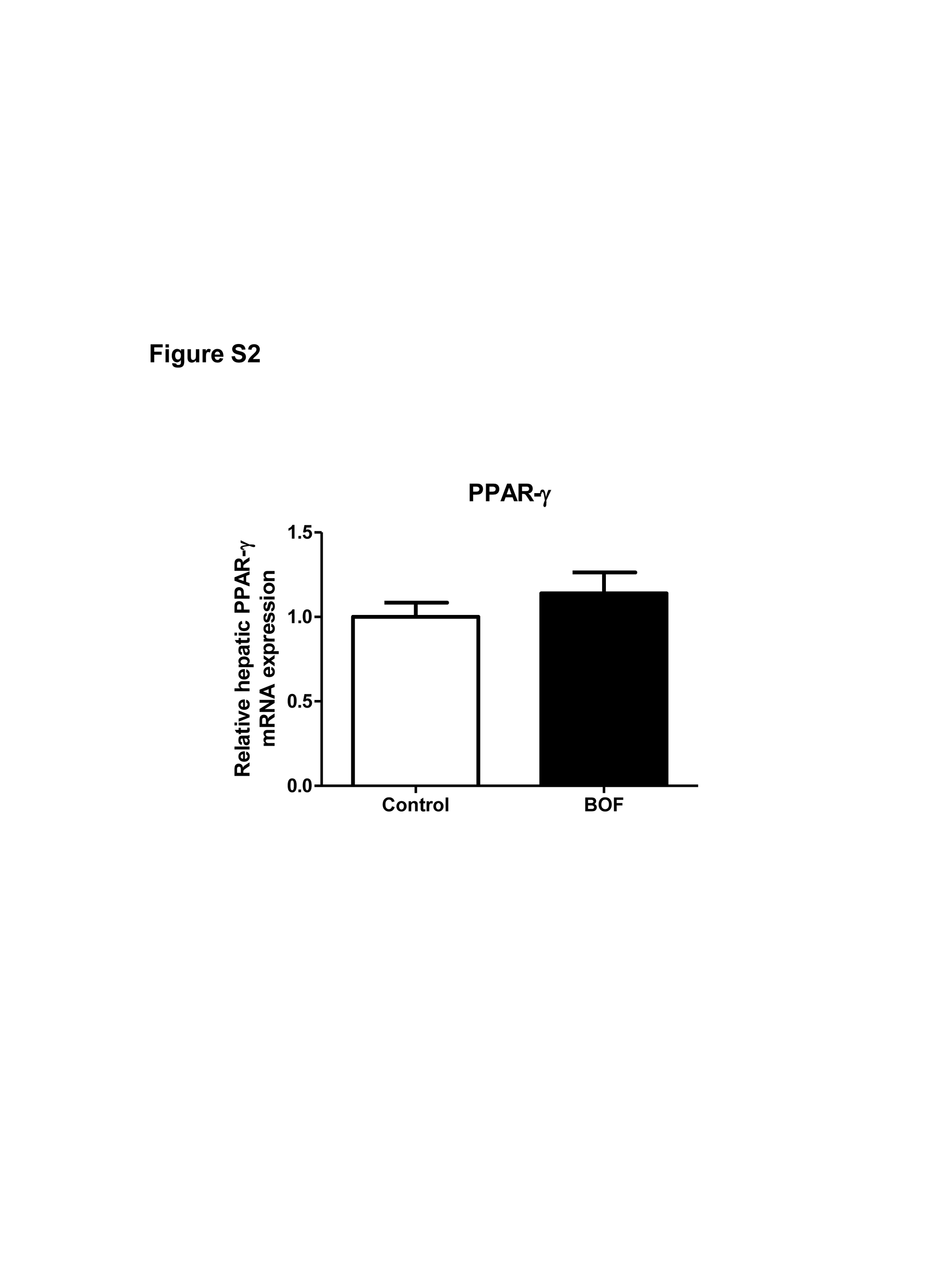

Supplement: Figure S2 — Chronic effects of BOF on PPAR-γ expression in liver. Expression of PPAR-γ mRNA in liver. Values are presented as the means ± SEM, *P<0.05 by Student’s t-test, vs. control group (n = 8). (TIF) [file pone.0075560.s002.tif]
